# Development of Alcohol Control Policy in Vietnam: Transnational Corporate Interests at the Policy Table, Global Public Health Largely Absent

**DOI:** 10.34172/ijhpm.2022.6625

**Published:** 2022-06-12

**Authors:** Sally Casswell

**Affiliations:** SHORE & Whariki Research Centre, College of Health, Massey University, Auckland, New Zealand.

**Keywords:** Global Health, Commercial Interests, Alcohol Policy, Cost Effective, Alcohol Reform Legislation, Vietnam

## Abstract

**Background:** This paper analyses input from global interests in the policy process leading up to the passing of alcohol control legislation in Vietnam in 2019. The global alcohol industry now relies on growth in volume in emerging markets in middle-income countries such as Vietnam, a large, rapidly industrialising country with a youthful population and emerging middle class. The industry’s role in the alcohol policy process is compared with that of global health interests.

**Methods:** Document analysis of letters and English language media coverage was supplemented by and triangulated with data from key informants on changes in the content of draft alcohol legislation and participant observation.

**Results:** The alcohol legislation was negotiated in the context of active engagement from the global alcohol industry and some input from global public health interests. The global alcohol industry established a partnership relationship with politicians using CSR and funded a local employee in Hanoi over the decade prior to the draft legislation being considered. Direct lobbying took place over the content of the legislation, which went through six published drafts. Trade and investment agreements provided a supportive environment and were referred to by both politicians and industry. In contrast public health resource was limited and lacked the support of a normative global policy to counter the economic imperatives. Vietnamese Ministry of Health proposals for cost effective alcohol policy were not enacted.

**Conclusion:** Global commercial interests employed their considerable resources to engage in corporate social responsibility (CSR) and build partnerships with policy-makers over a long period, contributing significantly to an environment unsupportive of enacting effective alcohol control policy. The absence of structural support from a global health treaty on alcohol and lack of an equivalent level of long-term sustained input from global health actors contributed to the legislative outcome, which excluded proposed cost-effective policies to reduce alcohol harm.

## Background

**Key Messages**

** Implications for policy makers**

- The policy holder for alcohol, often the health sector, needs to work with other government sectors, particularly economic sectors, to increase awareness of their responsibility in relation to alcohol attributable harm.
- National and local governments and non-governmental organisations (NGOs) need to establish clear terms of engagement with the alcohol industry and avoid all partnerships in corporate social responsibility (CSR) activities, given the conflict of interest that leads to the subverting of effective policy if industry participation in policy development is legitimised.
- The World Health Organization (WHO) secretariat could provide capacity building support to counter disinformation on health, social and economic costs and support regulation of commercial interests.
- International NGOs and philanthropists need to recognise that supporting effective alcohol policy is essential and actively support advocates and the health sector.
- The Member States of the WHO must provide a legal treaty to support alcohol policy similar to the Framework Convention on Tobacco Control (FCTC).

** Implications for the public**

Many governments, particularly in countries where alcohol products are being increasingly marketed and made readily available, are failing to regulate the alcohol industry to protect the population. Alcohol is a harmful product. It is addictive, carcinogenic and its use, often in heavy drinking occasions, results in harm to the family and community of the drinker, including family violence and traffic crashes. Identifying the role of the alcohol industry in undermining attempts by health officials to put in place effective policy may make people more aware of the possible dangers of partnerships between representatives of the alcohol industry and governments, as well as encourage governments to take a position independent of industry interests. Governments, including the ministries promoting economic development and trade, will then be encouraged to work collaboratively with the health sector and prioritise people’s health and wellbeing by ensuring that regulation effectively controls sale and supply, including marketing and affordability of alcohol products.

An emerging research literature over the past decade has documented the role of global alcohol industry influence in policy development in the context of neoliberal political ideology. Previously published case studies of the influence of vested interests on alcohol policy are mainly from high income countries and many relate to specific elements of industry practices, eg, response to research^1^ or specific elements of policy, for example, drink driving regulation,^2^ Minimum Unit Price^3^ or taxation,^4^ and advertising.^5^

 This Vietnamese case study adds to previous research by providing an in-depth analysis in a low- and middle-income country (LMIC) that has transitioned from a planned to a market led economy. It illustrates the contrasting role of global health entities and the global alcohol industry. It documents the industry response to an attempt by the domestic health sector to enact comprehensive alcohol control legislation.

###  Vietnam: An Expanding Market for Transnational Alcohol Corporations 

 Vietnam is a large, rapidly industrialising country with a government open to privatising the economy, a youthful population and emerging middle class, and low prevalence of drinking among women and younger people.^6^ It thus presents a major opportunity for expansion by the global alcohol industry.

 Commercial beer production was initially under state control in the Socialist Republic of Vietnam. However, in the context of reforms introduced to transition to a market economy, transnational alcohol corporations (TNACs) participated in the privatisation of production. Joint ventures, beginning in 2006, were followed by gradual investment. By 2018, around 90% of Vietnam’s beer market was controlled by the top four breweries, Sabeco (controlled by Thai Bev), Habeco (controlled by government), Heineken and Hue Brewery (owned by Carlsberg), while new players including AB InBev shared the remaining 10%.^7^

 Marketing is essential for the expansion of commercial alcohol brands. Prior to the development of the proposed alcohol reform legislation, marketing of beer was not regulated and beer was widely promoted. Marketing of wine and spirits was subject to some legal controls, however, there was extensive marketing at point of sale, and through sponsorships, product placement and social media.^8^

 Since 1990, per capita consumption of alcohol in Vietnam has increased by 90%, the largest observed increase in any large country over the time period.^9^ Beer sales increased by 43% from 2012 to 2017.^10^ It is projected that failing the implementation of effective alcohol control policy, consumption of commercial alcohol will increase further.^9^

###  Alcohol Policy Developments 

 Health officials in Vietnam were well aware of the consequences of an expanding alcohol market and the need for new legislation. A 2005 report highlighting increasing alcohol harms including hospitalisation, traffic crashes, social harms and public disorder recommended a minimum purchase age of 19 years and a ban on all forms of marketing of beer and other alcohol.^11^

 In 2012, a draft alcohol control Bill was introduced and expected to be presented to the National Assembly in 2016. However, a requirement for a policy impact assessment was also introduced in 2016, delaying the alcohol reform legislation. In the same year, a notable champion of alcohol policy, Dr. Nguyen Van Tien, Vice Chair of the National Assembly Social Affairs Committee, retired, while several previous champions of alcohol control policy had a change of heart and spoke in support of industry positions (Johan Bengtsson, personal communication).

 In 2017, the National Assembly agreed to put an alcohol Bill on the timetable for 2018. The Ministry of Health was assigned by the Government to take the lead in drafting the Bill, with the participation of representatives from responsible ministries and ministry-level organisations. The Bill, which was drafted with some assistance from the World Health Organization (WHO), was put out for public consultation, including across all responsible ministries and organisations, on April 5, 2018. The draft Bill went through six iterations before being adopted by the National Assembly in 2019.

 This analysis examines the influences shaping the final content of the legislation in the decade leading up to its enactment in 2019.

## Methods

 This paper presents a descriptive analysis of the alcohol policy development process in Vietnam.

###  Data Sources 

 Data sources included workshop reports, English language media coverage and letters to politicians during the course of policy development. The author participated in workshops on alcohol policy with Vietnamese government officials and the WHO.

 An early data source was the report of a workshop titled ‘Sharing Experiences for the Development of the National Policy on Alcohol-related Harm Prevention and Control,’ held 18–19 March 2009 in Hanoi, Socialist Republic of Vietnam, and organised by the Health Strategy and Policy Institute (HSPI) of the Ministry of Health in cooperation with the International Center for Alcohol Policies (ICAP). In addition, PowerPoint presentations shown at the workshop were provided by local key informants.

 Examples of letters from industry sources were provided by local key informants. These comprised:

1. Three letters in Vietnamese from Heineken Vietnam, including one dated May 21, 2018 to the Prime Minister, Vice Prime Minister, six ministers of responsible ministries, and the Chairman of the Government Cabinet Office. Two other letters (August 20, 2018; March 23, 2019) were distributed at meetings considering draft legislation. An English translation of the letter dated August 20, 2018 included Heineken’s recommendations for changes to the fourth draft of the Bill.
2. A letter in English from EuroCham (European Chamber of Commerce in Vietnam) and the Wine and Spirits Sector Committee to the Minister Health with detailed recommendations on the first draft of the Bill, dated March 31, 2018. The letter included an Appendix D entitled: ‘The Shortcomings of the Thai Health Promotion Foundation.’
3. A second letter in both Vietnamese and English from EuroCham and the Wine and Spirits Sector Committee to the Minister Health, dated January 8, 2019.
4. Letters in English from international civil society organisation the Global Alcohol Policy Alliance (August 7, 2018 and October 8, 2018) to the Prime Minister, Deputy Prime Minister, Minister of Health and the chairwomen of the National Assembly and Social Affairs Committee of the National Assembly, and an open letter from IOGT International (now Movendi International), August 24, 2018.^12^
5. A copy of the Questions and Answers document published by WHO.^13^

English language media coverage of the alcohol policy process was obtained by carrying out online (Google) searches using the following keywords: Vietnam, alcohol, beer, spirits, wine, legislation, and then following links to relevant media articles. In a few cases media links were provided by local collaborators. Twelve media reports were reviewed, covering the time period 2017–2020.

 Local key informants came from government, WHO and the non-governmental organisation (NGO) sector. They provided a summary of changes in the six versions of the Bill and reviewed drafts of the paper.

###  Analysis

 The report of the HSPI/ICAP meeting, PowerPoint presentations, letters from industry sources, and quotations regarding the policy (all from industry and politicians) published in the media sources were subjected to textual analysis using a critical interpretive approach that placed meanings attributed by both producers and audiences at the centre of analysis, along with likely effects.^14^ The comprehensive framework of corporate practices and mechanisms identified by Madureira Lima and Galea^15^ was used to guide the interpretation of meaning and effects.

 Local key informants identified the changes in the six published versions of the draft Bill and these were considered as objective sources identifying the facts of the policy process.^16^ Changes over time in the published versions of the draft Bill were analysed in relation to the inclusion or exclusion of the most cost-effective ‘best buys’ of alcohol policy.^17,18^

 The author was a participant in the workshop in Vietnam organised by HSPI in 2009 and participated as an invited technical expert in meetings organised by WHO, with support from Thai Health, at which Vietnamese participants presented data (November 9-10, 2009). The author also participated in a meeting with politicians organised by WHO in 2019 prior to the enactment of the legislation and received a copy of the publication ‘Questions and Answers.’ She also met with NGO representatives during visits to Vietnam.

 Local key informants clarified information and reviewed drafts of the paper for accuracy.

## Results

###  Weakening of the Legislation 

 This initial draft Bill (Version One) included several policies in line with WHO recommendations on the most cost-effective alcohol control policies.^18^ These included establishing trading hours, banning selling of any alcohol beverages via the Internet and increases in excise tax. Regulation of marketing was an important element. Some advertising of beer was allowed in this draft, however, it included content restrictions, a time restriction on TV advertising for beverages with potency less than 15% (beer and wine), a ban on alcohol sponsorship, and a ban on promotion in social media. A key element in Version One was establishing a health promotion agency through a tax based on a gradually increasing percentage of sales (to 2% in January 2030) to fund prevention activities (such a model had been established for tobacco in 2012).

 The Vietnamese legislation went through six published drafts. Version One was open for public comment and the five subsequent versions were revised by the responsible government bodies and then considered by the National Assembly’s Standing Committee. Version Six was finally approved by the National Assembly in June 2019. The drafts show a trend towards exclusion of cost-effective policy.

 Version Two saw the removal of trading hour restrictions as well as removing the proposed ban on selling beer via the Internet. Restrictions on marketing were weakened. An (ultimately unsuccessful) attempt was made to change the name of the law to refer to ‘alcohol abuse’ rather than ‘alcohol-related harms.’

 Version Three excluded a health promotion agency (but reintroduced the ban on internet sales of beer).

 Version Four removed the proposed ban on internet sales of beverages above 15% alcohol content.

 Version Five permitted the sale of all alcohol via the Internet, removed the proposed restriction preventing the use of company names and logos as part of sponsorships, and removed restrictions on alcohol being provided as part of promotions and competitions.

 Version Six, the final legislation, added zero tolerance for alcohol use when driving and increased penalties for drink driving.

 Responses to the Vietnamese Ministry of Health’s interest in achieving alcohol control policy legislation came from global vested interests and, to a lesser extent, public health prior to the drafting of the 2018 Bill. In 2009, the Western Pacific Regional Office of WHO funded a WHO Collaborating Centre to carry out a project on ‘Sustainable Alcohol Policy’ in four Mekong countries including Vietnam. A workshop held in Hanoi in November 2009, in collaboration with the Health Policy and Strategy Institute of the Ministry of Heath, provided technical input on the most effective policy to be included in legislation.^19^ However, before this initial public health workshop took place, the TNACs had commenced a targeted influence campaign.

 In 2008, representatives from Vietnam had attended a multi-stakeholder ‘Asia-Pacific Alcohol Forum’; this was hosted by the TNACs’ global public relations organisation, ICAP. In collaboration with Diageo, ICAP then co-hosted a workshop with the Ministry of Health’s Health Policy and Strategy Institute in 2009.^20^ The key message from the industry presenters was that, first and foremost, industry representatives must be partners in the development of alcohol policy; Diageo described the industry as ‘a key stakeholder.’ ICAP’s representatives recommended an emphasis on personal responsibility.^21^ ICAP team members were among those who travelled around the continent of Africa (another important market for expansion) promoting a model industry-friendly alcohol policy.^22^ In the years following, Brett Brivens, an ICAP employee, returned to Hanoi several times to work with local companies and participate in a number of workshops (pers. comm. Nguyen Phuong Nam). ICAP also employed a local country manager who had previously worked with the Global Road Safety Partnership.

###  Input to Legislation by Vested interests 

 The vested interests active in the policy process reflected the globalised nature of the alcohol industry. Four major TNACs were involved (Heineken, Diageo, Pernod Ricard and ABInBev). Two PR agencies representing the big global players in wine and spirits were also involved: the Vietnamese Alliance for Responsible Drinking, which was established in 2015 as a partner of the International Alliance for Responsible Drinking (IARD) (which by then had replaced ICAP as the major global industry PR body); and the Asia Pacific International Spirits & Wine Association. The European Chamber of Commerce (EuroCham), representing large exporters of wines and spirits, also played an active role.

 Examples of political tactics such as direct lobbying and preference shaping via corporate social responsibility (CSR) and attention deflection^15^ were observed in the process of policy development.

###  Direct Lobbying

 Criticisms of the draft Bills were framed within a partnership model: the August 2018 letter from Heineken stated their support for the Government’s objectives to ‘reduce abuse […]’ because abuse ‘threatens our sustainable business.’ However, in the same letter they advocated for numerous changes to the draft Bill, including allowing marketing of beer in outdoor media, sports, drama, movies, and the Internet, and allowing the selling of beer from automatic vending machines and via the Internet. Heineken also recommended alcohol branding of sponsorship should be allowed in the media; however, they argued health warning labels and setting trading hours should not be allowed.^23^

 Many letters were sent to National Assembly committees, local delegates and representatives of the National Assembly outlining concerns and detailed recommendations for changes in the legislation. EuroCham’s letter of January 8. 2019 referred in positive terms to the ‘transparency and openness’ shown throughout the process of drafting the law, with the participation of various relevant stakeholders. In this letter they argued against health warnings, the proposed ban on internet sales of alcohol, and the proposed ban on branding of sponsored events.^24^ This stance was supported by letters from six European wine and spirits companies, who wrote directly to Provincial Senator delegates to the National Assembly.

###  Funding/Donating

 Heineken worked closely with government and tied its support for a drink driving campaign to expenditure on marketing its products: the company promised to earmark at least 10% of its media spend on persuading Vietnamese consumers not to drink and drive.^25^

 Key informants reported the red envelopes, traditionally given to meeting participants and containing funds to cover meeting expenses, contained more money than usual at industry hosted workshops attended by journalists and policy-makers.

 After the proposal to introduce trading hours had been rejected by the National Assembly, members were asked in a media conference if interest groups had influenced the draft legislation, given reports some lawmakers had travelled abroad at the invitation of the alcohol companies. While denying influence, the deputy head of the Committee on Social Affairs stated only ‘a few, if any, joined the businesses survey trips*.’*^26^

###  Trade and Investment Agreements

 Many of the arguments made by the industry against the legislation were aligned with Vietnam’s aspirations as an economy open to transnational corporations and committed to economic development.^24^ Reference to Vietnam’s membership of trade and investment treaties was used to justify and support industry friendly policies by both politicians and industry. References to trade agreements were made by politicians when rejecting controls on trading hours.^26^ And in arguing against the health promotion agency and in favour of CSR, Heineken referred to the Vietnam-European Union (EU) free trade agreement: ‘In fact, beer producing enterprises are doing effectively ‘corporate social responsibilities … in line with Vietnam’s commitment in Vietnam – EU Free Trade Agreement.’^23^ Heineken also claimed restrictions on advertising were in contradiction to the country’s Asia-Pacific Economic Cooperation commitments.^27^

###  Importance of Global Business and Economic Interests 

 Policy-makers and TNACs expressed strong sentiment in favour of industry investment, with the Prime Minister asking Carlsberg to increase its role in Vietnam in the interests of economic cooperation between Europe and Vietnam, and in line with the Vietnam-EU free trade agreement. Carlsberg talked about its relationships with the Ministry of Trade and Industry and ‘hoping that its business operations in Việt Nam will become more and more effective.’^28^

 Economic benefits from the alcohol and advertising industries were a major theme. Relying on business to promote responsible drinking was justified as an economic saving.^29^ Speaking at a WHO/Ministry of Health workshop shortly before the Bill was passed, the Vice Chairman of the Social Affairs Committee acknowledged adverse health and social consequences from alcohol but said economic interests were also important and the alcohol industry brought taxes and employment.^30,31^

###  Establishing Partnership Via Corporate Social Responsibility

 Delays in initiation of the alcohol reform legislation allowed for several years of active engagement between vested interests and government via CSR.

 CSR activities were an important element in building partnerships and influencing policy options. Throughout the ten years leading up to and during the negotiation of the alcohol reform legislation, the TNACs and trade organisations partnered with the high-level government committee of the National Traffic Safety Agency (NTSA). The many workshops held to showcase industry involvement in educational activities and drink driving campaigns^32^ were covered in the media. For example, a 2018 workshop co-hosted by the NTSA Committee, Asia-Pacific International Spirits and Wine Alliance and Pernod Ricard was reported on in the central organ of the Communist Party, Nhan Dan, including comments made by the Deputy Head of the Office of the NTSA Committee applauding the work on drink driving by Asia Pacific International Wine and Spirits Alliance (APIWSA).^29^ The influence of CSR on the development of policy was illustrated by the Vice Chairman of the National Assembly’s comment about APIWSA’s CSR activities: ‘This is a basis for the Vietnamese National Assembly to study mechanisms and policies on wine, beer and alcohol drinks.’^33^

 The alcohol industry referred to their CSR activities in their arguments against the establishment of a health promotion agency, which was proposed in the first draft of the Bill. Their argument that such an agency would prevent them carrying out CSR activities was reported in the media under the headline‘Draft law to prevent alcohol firms’ social responsibility programmes,’^34^ illustrating the way CSR activities can be used to avoid the formalising of informal measures into statutory regulation.^15^

###  Issue Framing/Attention Deflection

 A common approach to deflecting attention away from regulation of commercial alcohol products, especially in LMICs, is to encourage a focus on non-commercial alcohol. EuroCham stressed the considerable contribution non-commercial alcohol makes to Vietnamese alcohol consumption and referred to an IARD funded survey of informal alcohol use.^35^ It went on to claim consumers of non-commercial alcohol were more likely to drink heavily. However, survey data from the International Alcohol Control study suggests commercial alcohol is more likely than non-commercial alcohol to be consumed in heavier drinking occasions in Vietnam.^36^

 A common aspect of CSR globally is to promote education with a ‘drinking responsibly’ message,^37^ a framing that deflects attention away from industry practices such as marketing and supply. However, such campaigns have considerable marketing potential in contexts with relatively low consumption among the young as they normalise alcohol use among the young and also reinforce its role in western culture.^15,38^ In workshops held in co-operation with Ho Chi Minh City Youth Union^39^ as part of ABInBev’s Smart Drinking Goals campaign,^40^ university students were told: ‘Beer and other alcoholic drinks are part of social etiquette in Vietnam and around the world*.’*^39^

 Voluntary codes on alcohol marketing, referred to by the industry as ‘self-regulation,’ are also commonly used to justify opposition to any regulation of marketing and a voluntary code already established by industry interests in Vietnam was used to argue against provisions in the draft Bill to regulate alcohol marketing.^41^

###  Manufacture of Doubt/Inaccurate Information 

 The campaign to prevent the establishment of a health promotion agency also manufactured doubt regarding its likely effectiveness.^15^ In a letter from Heineken to National Assembly members it was claimed a small number of countries had established this approach but ‘almost all … have been abolished … or shown no clear effectiveness.’ Heineken and the European Chamber of Commerce^41,42^ alleged wrong doing and ineffectiveness on the part of Thailand’s health promotion agency, Thai Health, which had been held up as a valuable model for Vietnam and had also co-funded capacity building activities in the region with the WHO.

 Industry spokespeople were presented as experts in the media and, in response to the proposal in the draft legislation to introduce trading hours for alcohol sale, a policy supported by research,^43-46^ industry representatives, including the director of foreign affairs for Heineken, claimed this policy would not work: ‘Vietnam’s overnight alcohol ban proposal unfeasible: experts.’^47^

 A local industry trade organisation, the Vietnam Beer and Alcohol Beverage Association, denied the causal relationship established between alcohol and cancer,^48,49^ referring to a Vietnamese Ministry of Health statement regarding alcohol and cancer as ‘controversial’ and claiming that ‘according to a scientific report 2018 of World Cancer Research Fund, only breast cancer is related to alcohol drinking but at low risk level.’ This paper goes on to state alcohol is ‘not harmful to health but even good for health.’^50^

###  Global Health Entity Input 

 Public health advocacy to support the Ministry of Health’s efforts came primarily from the country and regional offices of WHO, and by means of letters to politicians from international agencies including the Global Alcohol Policy Alliance and Movendi. Several meetings in the region were organised by the WHO Western Pacific Regional Office (with support from Thai Health), which also sponsored two or three Vietnamese personnel to attend regional meetings to discuss effective alcohol policy. The WHO country office provided support throughout the process including, with the IOGT-NTO Movement, help in the initial drafting of the legislation, but much of their effort was focused on the period leading up to the final decision on the legislation. A Questions and Answers document was published in 2019^13,51^ and an overseas adviser (the author) invited to speak at a meeting with politicians. Local branches of the international NGOs Healthbridge and NCD Alliance were active in the debate. Vn NCDA sent letters to politicians and reached out to international NGOs, the Global Alcohol Policy Alliance and Movendi, which both wrote letters in support of the need for evidence-based policy. The Global Alcohol Policy Alliance’s second letter in October 2018 specifically addressed the weakening of the draft legislation, particularly in relation to marketing regulation and the challenges to the establishment of a health promotion agency. Both Movendi and the Global Alcohol Policy Alliance referred to the Bloomberg-WHO analysis, which estimates a saving of US$9 for every $1 spent on effective alcohol policy.

## Discussion

 Analysis of the policy process from the data sources available illustrated a very large imbalance of resource investment and engagement by the vested interests compared with the public health sector, with a concomitant impact on policy development. Changing iterations of the draft legislation reflected the advocacy by the vested interests to exclude the most cost-effective policies from the legislation and allowed internet sales of all beverages and continued marketing of beer and wine. The failure to secure the proposed restrictions on logos and brands in sponsorship, for example, allowed the launch of Formula One branded by Heineken in Vietnam.

 The political context enabling the influence of TNACs on alcohol policy started in the mid-1980s when Vietnam adopted ‘Doi Moi,’ an economic renovation policy that included the promotion of the production of consumer goods, reduction of State intervention in business, and encouragement of foreign private investment.^52^ As part of Vietnam’s increasing participation in a globalised free-market-led political economy, the involvement of corporations with huge market power and resources was welcomed by policy makers. However, the response to the Ministry of Health’s efforts to put in place effective alcohol policy stands in stark contrast to Vietnam’s record on tobacco control. Following the ratification of the Framework Convention on Tobacco Control (FCTC) in 2005, Vietnam established the Vietnam Tobacco Control Fund, which is funded by a compulsory contribution from the tobacco industry. Further, despite ongoing interference from the industry, this fund has succeeded in building capacity and building new infrastructure for tobacco control, including a total ban on tobacco marketing.^53^

 This process of policy development in Vietnam illustrates the imbalance between TNAC resources and their purposeful long-term engagement in countries targeted for market expansion, compared with the resources available to promote effective alcohol control by global health entities. Heineken, Diageo, Pernod Ricard and ABInBev, with a combined annual global revenue of approximately US$93.5 billion, were all active in Vietnam, as were industry alliances such as IARD, APIWSA and EuroCham. ICAP, the precursor to IARD, funded a local employee in the years prior to the legislative process. Their campaigns reflected the importance of Vietnam to market expansion, and as a model for other Mekong emerging markets. The resources available to support health interests were very much smaller and regional and international input was irregular and insufficient. There was no influence from global health entities equivalent to the well-resourced public-private partnerships or long-term relationships crucial to their successful lobbying that the industry built with members of the National Assembly.

 The Ministry of Health officials worked hard with the country office of WHO, enlisted informal support from international scientists and NGOs, and spoke out about the weakness of the legislation before it was passed.^54^ However, as seen so often in the development of hotly contested policy in relation to regulating unhealthy commodities, the structural reality of Vietnam’s political economy meant the health sector’s political influence was considerably less than that of the economic sector.^55^

 Many of the corporate practices identified by Madureira Lima and Galea^15^ were observered in this LMIC setting. The industry succeeded in keeping conflict mostly latent through their long-term development of a ‘partnership’ framing, applauding government efforts, and providing cohesive and consistent messaging in their public statements that undermined acceptance of the evidence-based policies. The inclusion of drink driving policy in the final version of the draft Bill (following two fatalities in Hanoi and public protest^51^) served to distract attention from the loss of much cost-effective policy.^56^

 Trade and investment treaties and other international agreements, which supported the Vietnam government’s commitment to transition from a socialist to a market economy, were frequently referred to by both industry interests and politicians; the chilling effect of trade treaties^57^ was apparent. In contrast, there was no reference in the media or from politicians to any global normative statements on alcohol policy. The non-binding WHO Global strategy on reducing harmful use of alcohol, in place during the policy process, had no visibility and no apparent impact. This is in line with findings from trade treaty negotiations in which the binding health treaty, the FCTC, played an important normative role in trade discussions about tobacco while the global strategy on alcohol was largely invisible.^58^

 This case study of Vietnam provides a further example of successful industry efforts to shape alcohol policy to prevent any threat to their sales and profits.^59^ Industry profits rely on heavy drinking^36^ and effective policy reduces heavy drinking. Unlike the global response to the conflict of interest of the tobacco industry, where Clause 5.3 of the FCTC specifically requires governments to prevent industry influence on policy development, there was no equivalent protection for the development of alcohol control policy in Vietnam.

 Asia remains a major focus for the TNACs. The same pattern of engagement is visible in other potential growth markets such as Cambodia^60,61^ and Myanmar.^62^ The TNACs are well underway with building the long-term relationships that will serve their interests and the global health governance field has so far remained unwilling to attempt to disrupt their business practices in the interest of protecting health.^63^

###  Limitations

 Submissions to the first public consultation were not made available and only English language documents and translations were able to be understood by the author. Key informants translated and clarified any uncertainties. The analysis relied largely on information contributed by key informants and media coverage, but other influences on and reasons for policy decisions were not accessible.

## Conclusion

 This case study illustrates industry influence contributing to much less effective alcohol control policy than that originally proposed by the government health sector. The industry developed a close, long-term engagement with policy-makers using CSR that involved public-private partnerships. In direct lobbying, the industry manufactured doubt and framed arguments to deflect attention away from their practices. The input from global transnational corporations and related organisations with huge resource; the supportive economic agreements, reference to which was used to chill the uptake of effective policy; the absence of any global health treaty providing normative support for effective control policy and protection against industry influence; and the lack of adequate and timely public health resource all contributed to a failure to protect the Vietnamese population from continued expansion of the alcohol market and consequent increases in alcohol harm.

## Acknowledgements

 I am grateful to Nguyen, Phuong Nam of the WHO Vietnam Office, Johan Bengtsson, from IOGT, Sweden, and to Vietnamese informants, who preferred to remain anonymous, for their support in the preparation of this paper. Participant observation was based on the author’s role as a technical advisor to WHO WPRO. I am grateful to the WHO Country Office staff in Vietnam who facilitated my visits in 2009 and 2019.

## Ethical issues

 The project was conducted under Massey University research and ethical procedures. In line with this, the research was assessed by peer review to be low risk. Consequently, it was not reviewed by one of the University’s human ethics committees, and the researcher (author of this article) was responsible for the ethical conduct of the research. Upon their request, the key informants who provided translated documents, information and checked paper drafts for accuracy remained anonymous.

## Competing interests

 Author declares that she has no competing interests.

## Author’s contribution

 SC is the single author of the paper.
